# Mitochondrial Deficits With Neural and Social Damage in Early-Stage Alzheimer’s Disease Model Mice

**DOI:** 10.3389/fnagi.2021.748388

**Published:** 2021-12-10

**Authors:** Afzal Misrani, Sidra Tabassum, Qingwei Huo, Sumaiya Tabassum, Jinxiang Jiang, Adeel Ahmed, Xiangmao Chen, Jianwen Zhou, Jiajia Zhang, Sha Liu, Xiaoyi Feng, Cheng Long, Li Yang

**Affiliations:** ^1^Precise Genome Engineering Center, School of Life Sciences, Guangzhou University, Guangzhou, China; ^2^School of Life Sciences, South China Normal University, Guangzhou, China; ^3^South China Normal University-Panyu Central Hospital Joint Laboratory of Translational Medical Research, Panyu Central Hospital, Guangzhou, China; ^4^South China Research Center for Acupuncture and Moxibustion, Medical College of Acu-Moxi and Rehabilitation, Guangzhou University of Chinese Medicine, Guangzhou, China

**Keywords:** Alzheimer’s disease, hippocampus, medial prefrontal cortex, mitochondrial dynamics, social interaction

## Abstract

Alzheimer’s disease (AD) is the most common neurodegenerative disorder worldwide. Mitochondrial dysfunction is thought to be an early event in the onset and progression of AD; however, the precise underlying mechanisms remain unclear. In this study, we investigated mitochondrial proteins involved in organelle dynamics, morphology and energy production in the medial prefrontal cortex (mPFC) and hippocampus (HIPP) of young (1∼2 months), adult (4∼5 months) and aged (9∼10, 12∼18 months) APP/PS1 mice. We observed increased levels of mitochondrial fission protein, Drp1, and decreased levels of ATP synthase subunit, ATP5A, leading to abnormal mitochondrial morphology, increased oxidative stress, glial activation, apoptosis, and altered neuronal morphology as early as 4∼5 months of age in APP/PS1 mice. Electrophysiological recordings revealed abnormal miniature excitatory postsynaptic current in the mPFC together with a minor connectivity change between the mPFC and HIPP, correlating with social deficits. These results suggest that abnormal mitochondrial dynamics, which worsen with disease progression, could be a biomarker of early-stage AD. Therapeutic interventions that improve mitochondrial function thus represent a promising approach for slowing the progression or delaying the onset of AD.

## Introduction

Alzheimer’s disease (AD) is a progressive, multifactorial, age-dependent, neurodegenerative disorder characterized by loss of memory, impairment of cognitive and non-cognitive functions, and changes in personality and behavior (Saez-Atienzar and Masliah, 2020). At the cellular and molecular levels, AD is associated with loss of neurons, neurofibrillary tangles, and amyloid β (Aβ) deposits in the cortex and hippocampus (Van Der Kant et al., 2020). However, several recent fundamental discoveries highlight important pathological roles for other critical cellular and molecular processes. Despite this, no disease-modifying treatment currently exists, and numerous phase 3 clinical trials targeting Aβ have failed to demonstrate benefit.

The mitochondrion, known as the powerhouse of the cell, is the organelle that produces the energy (in the form of adenosine triphosphate, ATP) necessary for the survival and optimal function of neurons (Spinelli and Haigis, 2018). Multiple studies suggest that mitochondrial ATP levels in affected brain regions of AD patients and mouse models are reduced (Reddy, 2008; Yao et al., 2009; Terni et al., 2010; Cha et al., 2015; Gauba et al., 2019). Neurons are especially vulnerable to mitochondrial dysfunction due to their high energy demand and dependence on the respiration for ATP generation. Mitochondrial dysfunction increases reactive oxygen species (ROS) production, leading to oxidative stress, neuroinflammation (Joshi et al., 2019), and subsequent neuronal damage in AD (Wang et al., 2020; Misrani et al., 2021). Despite evidence suggesting that mitochondrial dysfunction (Hauptmann et al., 2009; Wang et al., 2020) and abnormal synaptic transmission (Selkoe, 2002; Styr and Slutsky, 2018) are early events, earlier than the appearance of Aβ plaques in AD pathology, which comes first has not been evaluated side-by-side.

The prefrontal cortex (PFC) and hippocampus (HIPP) brain regions, which are critically involved in cognition and decision making (Spellman et al., 2015), are among the earliest areas to suffer impairment during AD progression (Grady et al., 2001; Wang et al., 2006). The PFC governs many higher-order executive tasks such as learning, memory (Warden and Miller, 2010), cognitive flexibility (Gruber et al., 2010), and emotional processing (Parfitt et al., 2017; Koush et al., 2019). Abnormal PFC activity, such as impaired executive functioning and working memory, is reported in AD patients (Satler and Tomaz, 2011; Stopford et al., 2012). The HIPP is widely studied in AD as this brain region is essential for forming new memories, and the progressive degeneration of neurons in the HIPP is responsible for the short-term memory loss that is a hallmark of AD (Selkoe, 2002; Wei et al., 2010). Moreover, reduced numbers of dendritic spines and altered synaptic transmission in PFC and HIPP are early events in AD (Wei et al., 2010; Styr and Slutsky, 2018; Ammassari-Teule, 2020). The neuronal projections from the HIPP to the PFC, referred to as the HIPP-PFC-circuit, play a critical role in cognitive, social, and emotional regulation. Furthermore, altered functional connectivity of the HIPP-PFC circuit occurs in AD, leading to cognitive impairment (Zaidel et al., 2012; Xue et al., 2019). Nevertheless, although synaptic dysfunction and altered connectivity of brain circuits are characteristics of AD (Selkoe, 2002; Styr and Slutsky, 2018; Xue et al., 2019), whether AD pathogenesis alters the HIPP-mPFC pathway in APP/PS1 mice is unclear.

In this paper, we used amyloid precursor protein/presenilin 1 (APP/PS1) double transgenic mice (Jankowsky et al., 2001) (1) to determine the earliest age at which mitochondrial alteration occurs in this mouse AD model; (2) to explore whether neuronal morphological and synaptic dysfunction co-occur with mitochondrial dysfunction; (3) to evaluate whether the earliest defects affect social behavior, which requires normal HIPP-mPFC activity. The results, which reveal severe defects in mitochondrial dynamics together with neuroinflammation in the brains of young APP/PS1 mice, may stimulate the development of new therapeutic strategies for AD.

## Materials and Methods

### Animals and Housing

Amyloid precursor protein/presenilin 1 (APP/PS1) double transgenic mice, derived from the B6C3-Tg (APPswe, PSEN1dE9) 85Dbo/J strain (JAX 004462), which expresses a chimeric mouse/human APP gene (APPswe) and human mutant PS1 (DeltaE9), were maintained at the animal house facility of the School of Life Sciences, Guangzhou University. Both male and female APP^swe^/PS1^ΔE9^ and their WT littermate mice were used in this study. The mice were genotyped by polymerase chain reaction (PCR) according to the Jackson Laboratory (JAX) protocol. Animals were housed in cages where mice could eat and drink freely, with a 12-h light-dark cycle. All animals involved in experiments were 1∼2, 4∼5, 9∼10, and 12∼18 months old unless otherwise indicated.

### Western Blotting

Mouse brains were rapidly dissected on ice, and mPFC and HIPP tissues were homogenized in lysis buffer (50 mM Tris pH 7.5, 150 mM NaCl, 5 mM EDTA pH 8.0, 1% SDS and protease inhibitors (Complete Mini; Roche)). After centrifugation at 4°C (14,000 rpm for 10 min), cellular debris was removed, and the supernatant was collected for western blotting. Tissue lysates were analyzed by sodium dodecyl sulfate-polyacrylamide gel electrophoresis (SDS-PAGE) and separated proteins were transferred to nitrocellulose membranes. Membranes were then blocked with 5% defatted milk in Tris-buffered saline with Tween 20 (TBST) for 1 h and incubated overnight at 4°C with the following specific primary antibodies against Drp1 (Abcam, ab184247; dilution 1:1,000), Mfn1 (Abcam, ab57602; dilution 1:3,000), Mfn2 (Abcam, ab56889; dilution 1:3,000), OPA1 (Abcam, ab157457; dilution 1:2,000), ATP5A (Abcam, ab14748; dilution 1:3,000), PSD95 (Abcam, ab2723; dilution 1:3,000), GFAP (Thermofisher, 13-0300; dilution 1:1,000), Iba1 (Wako, 019-19741, dilution 1:1,000), nitrotyrosine (Santa Cruz: sc-32757; dilution 1:1,000), Cleaved caspase-3 (Abcam, ab13847; dilution 1:1,000) and Nrf2 (Abcam, ab137550; dilution 1:3,000). Anti γ-tubulin antibody (Sigma, T6557) was used as a loading control. After three washes with TBST, HRP-labeled secondary antibody (CWS, China) was added at room temperature for 1 h using 5% milk in TBST, followed by three additional washes with TBST. The Immobilon ECL western system (Millipore, United States) was then used to visualize the bands, which were quantified and analyzed with Gel-Pro Analysis software (Media Cybernetics, United States).

### Transmission Electron Microscopy (TEM)

The mPFC and HIPP were fixed overnight in the 2.5 % (v/v) glutaraldehyde and then in 1 % (w/v) OsO_4_ for 1 h. The fixed slices were dehydrated in an ascending series of ethanol finishing with absolute alcohol and embedded in EPON resin. Ultra-thin sections (70 nm thick) were cut using (LEICA UC7/FC7 Ultramicrotome) from the tissue slices that were stained with uranyl acetate and lead citrate and then examined under a JEM 1400 plus Japan transmission electron microscope (TEM) as described previously (Moriguchi et al., 2019). The mitochondrial ultra-microstructure of pyramidal cells in the mPFC and HIPP was examined under magnification of × 23,000 and × 49,000. The number of mitochondria and mitochondrial aspect ratio (length/width) were calculated using ImageJ software.

### Golgi-Cox Staining

The Golgi-Cox staining protocol followed a routine procedure (Zhang et al., 2020). All animals were coded before the Golgi-Cox method to blind the experimenter to the animal’s identity until the data analysis was completed. Briefly, animals were perfused transcardially with 4% paraformaldehyde (PFA). The brains were rapidly removed and stored in Golgi-Cox solution in the dark at room temperature (RT) for 14 days; the Golgi-Cox solution was refreshed every 48 h to remove sediments. These brains were then transferred into a 30% sucrose solution for 7 days, and the sucrose solution was changed every day. Brains were then embedded in paraffin wax, and 150 μm thick brain slices from mPFC and HIPP were cut using a microtome (Leica, Germany).

Medial prefrontal cortex and hippocampus pyramidal neurons were identified based on the following criteria: (1) location within the mPFC or HIPP; (2) staining of the intact neuron; (3) triangular-shaped soma and single axon; and(4) no direct contact with neighbouring neurons. All neurons were reconstructed under a light microscope (Nikon, Japan) using a 40× lens. Image J software was used to analyze dendritic length, the number of branches, and the morphological complexity of the cells.

### Electrophysiology

#### Whole-Cell Patch-Clamp Recording

Acute brain slices containing mPFC and HIPP (350 μm) were prepared according to routine procedures (Chen et al., 2017) from WT and APP/PS1 mice using a vibratome (VT 1000S, Leica, Germany) in an oxygenated ice-cold cutting solution containing (in mM), 119 NaCl, 2.5 KCl, 2.5 CaCl_2_, 1.3 MgSO_4_, 1 NaH_2_PO_4_, 11 D-glucose, 26.2 NaHCO_3_ (pH 7.2–7.4), saturated with 95% O_2_/5% CO_2_. Slices were kept in artificial cerebrospinal fluid (aCSF) containing (in mM) 140 NaCl, 4.7 KCl, 2.5 CaCl_2_, 1.2 MgCl_2_, 11 D-glucose, 10 HEPES (pH 7.2–7.4), and gassed with 95% O_2_/5% CO_2._ Slices were then incubated for 1 h at 30–32°C before recording and then transferred to a submerged recording chamber where the temperature was held at 32 ± 0.5°C with an automatic temperature controller (TC-324B, Warner Instrument Corporation) with aCSF flow set at 2–3 ml/min.

To record miniature excitatory postsynaptic current (mEPSC) and miniature inhibitory postsynaptic current (mIPSC) from pyramidal neurons of the mPFC, the voltage was held at −60 and 0 mV, respectively. To block fast sodium channel activity and action potential, 1 μM TTX was added to the aCSF. The pipette was filled with the following internal solution (mM): 100 mM Cs-gluconate, 5 mM CsCl, 10 mM HEPES, 2 mM MgCl_2_, 1 mM CaCl_2_, 11 mM BAPTA, 4 mM ATP and 0.4 mM GTP (pH 7.3, adjusted with KOH) at an osmolality of 280–290 mOsm. In another experiment using a Drp1 inhibitor named mitochondrial division inhibitor (Mdivi-1) (Huang et al., 2015; Baek et al., 2017), we asked if neuronal functional alterations could be prevented. Therefore, we recorded mEPSC of pyramidal neurons of mPFC in 4∼5-month-old APP/PS1 mice, treated with DMSO (Vehicle) or Mdivi-1 (Mdivi-1). Mdivi-1 (Sigma Aldrich, 475856-10MG) was prepared as a stock solution (10 mM) and was diluted with DMSO to the final concentration immediately before use. Brain slices were incubated with either Mdivi-1 at a final concentration of 10 μmol/L or DMSO for 1 h followed by the recording of mEPSC. DMSO or Mdivi-1 containing aCSF was continuously perfused over slices during recording. Data were collected with a MultiClamp 700 B amplifier (Axon Instruments) and filtered during acquisition with a low pass filter set at 2 kHz using pCLAMP10 software (Molecular Devices, United States). The data were analyzed offline using Mini Analysis Program (Synaptosoft Inc., United States).

#### *In vivo* Surgery and Local Field Potential (LFP) Recording

*In vivo* dual-site extracellular recordings were conducted as described (Engel et al., 2001). Mice were anesthetized with pentobarbital sodium (IP 80 mg/kg), then head-fixed in a stereotaxic apparatus (RWD Life Science) with body temperature maintained between 36 and 37°C. When necessary, a supplemental dose of anesthesia was given based on tail reflex. After a midline skin incision was made, two skull holes were drilled above the mPFC (1.98 mm anterior to the bregma, 0.5 mm lateral to the midline, 1.2 mm depth) and the CA1 subregion of the HIPP (−2.06 mm posterior to the bregma, −1.5 mm lateral to the midline, 1.0 mm depth) under a stereomicroscope (Sunny Optical Technology). Two glass microelectrodes for recording (filled with 0.5 M NaCl, with a resistance of 1.0–1.5 MΩ) were slowly inserted until the tips of the electrodes reached the mPFC and hippocampal CA1. Each recorded signal was amplified (1,000×) by an electrometer amplifier (Model 3000; A-M Systems) and digitized *via* a D/A converter (Micro 1401; Cambridge Electronic Design), then sent to data acquisition software (Spike2; Cambridge Electronic Design).

#### Local Field Potential Analysis

Local field potential data was analyzed offline in MATLAB 2012a (MathWorks) and spike2 (Chen et al., 2021). For processing the LFP, a Butterworth low pass filter (300 Hz) was applied to the raw recorded data. Synchronization was evaluated using cross-correlation analysis in line with our established protocol (Chen et al., 2019). Simultaneously recorded data were first low-passed using a third-order Butterworth filter in a phase-preserving manner and then subjected to normalized estimation of similarity. The maximal offset was set to ± 1 s for cross-correlation analysis. After the calculation, spectral coherence between the two LFPs from the mPFC and HIPP was analyzed under an FFT number of 512, and values were obtained in the range from 0 to 1, meaning non- (0) or completely (1) correlated in the frequency domain.

### Immunofluorescence

Mice were anesthetized by IP injection of 20% urethane (0.01 ml/g) and perfused transcardially with 0.9% saline and 4% paraformaldehyde (PFA) in PBS (0.01 M, pH 7.4). Immediately after perfusion, brains were dissected and post-fixed in 4% PFA for 24 h, then dehydrated by immersion in a 15% sucrose solution overnight followed by a 30% sucrose solution until the tissue no longer floated in the sucrose solution. Serial coronal/sagittal sections of mPFC and HIPP tissues were cut to a 30 μm thickness using a Leica CM30505 freezing microtome (Leica, Germany). The slices were then treated for membrane permeabilization with 0.5% Triton-X 100 and blocked with 5% BSA in 0.01 M PBS for 1.5 h at RT, followed by incubation with primary antibody against rabbit anti-Iba1 (Wako, 019-19741, 1:1,000) and anti-GFAP (Thermofisher, 13-0300, 1:1,000) overnight at 4°C in 1% BSA/PBS. The following day, slices were given three 10 min washes with PBS and incubated with secondary antibody (Invitrogen) at RT for 2 h followed by three additional washes with PBS for 10 min. Each section was imaged using a 20× lens under a fluorescence microscope (Nikon, Japan). Positive-cell counting was done using Image-Pro Plus (Media Cybernetics, United States) and Image J software (National Institutes of Health, Bethesda, MD, United States).

### Three-Chamber Social Interaction Test

Social memory can be quantified by measuring the relative interaction durations with a novel and a familiar mouse under free-choice conditions (social discrimination test or SDT) as described previously (Okuyama et al., 2016). Briefly, the three-chambered apparatus is composed of a solid rectangular plexiglas container (60 × 44 × 40 cm^3^) separated into three sections (each 20 × 44 × 40 cm^3^) by plain Plexiglas walls containing openings. An inverted cage (10.5 cm high × 10.5 cm diameter bottom × 7.6 cm diameter top, 1 cm bar spacing) was placed in each of the two outer chambers (ZhengHua Instruments, China). During a habituation period, the mouse could freely explore the apparatus for 10 min. Subsequently, in the test phase, a cagemate mouse was placed in one social chamber, whereas a stranger non-cagemate mouse of the same age and sex as the familiar mouse was placed in another social chamber. Then, the experimental mouse was permitted to explore the entire apparatus for 10 min. Duration of sniffing by test mice of each cage was recorded, and the discrimination index was calculated as follows: Duration _familiar_, total sniffing duration for cagemate mouse; Duration _novel_, total sniffing duration for stranger non-cagemate mouse).

$$\mathrm{Discrimination}\mathrm{index}=\frac{{\text{Duration}}_{\text{novel}}-{\text{Duration}}_{\text{familiar}}}{{\text{Duration}}_{\text{novel}}+{\text{Duration}}_{\text{familiar}}}$$

### Statistics

Prism 8.0 for Windows (GraphPad, United States) and OriginPro 2020 (OriginLab, United States) software were employed for graphing and statistical analysis. A two-sample *t*-test was used for statistical analyses between two-group comparisons. For the Golgi-Cox staining analysis, one-way ANOVA with a *post hoc* test was performed. Sholl analysis was performed using two-way repeated measure ANOVA; unless otherwise stated. *p* < 0.05 was considered statistically significant. The data are presented as mean ± SEM.

## Results

### Altered Levels of Mitochondrial Fission, Adenosine Triphosphate Synthase Protein and Mitochondrial Morphology in 4∼5 Month-Old Amyloid Precursor Protein/Presenilin 1 Mice

Mitochondria dynamics involve two specific, highly regulated opposing processes known as fission and fusion (Westermann, 2010; Kandimalla et al., 2016), which are fundamental aspects of mitochondrial biology and its quality control (Detmer and Chan, 2007). Mitochondrial fission requires the action of dynamin-1-like protein (Drp1), which is critical for mitochondrial division, size, shape and distribution throughout the neuron (Lee et al., 2016). In contrast, mitochondrial fusion requires the action of the mitofusin-1 (Mfn1) and mitofusin-2 (Mfn2) oligomeric complexes to tether the outer membranes of two fusing mitochondria (Chen et al., 2003). Inner membrane fusion is mediated by the inner membrane optic atrophy type 1 (OPA1) protein (Cipolat et al., 2004). Considering the crucial role of mitochondrial fission and fusion in neuronal function, and the occurrence of mitochondrial dysfunction in AD, we first sought to determine the earliest age at which any alteration in these fission/fusion proteins occurs in APP/PS1 mice. We evaluated mitochondrial fission, fusion, and ATP synthase protein levels in mPFC and HIPP of young 1∼2 month-old APP/PS1 mice. Our western blotting results of mPFC extracts revealed no significant difference in the levels of Drp1 (WT: 1 ± 0.31; APP/PS1: 0.87 ± 0.29; *p* = 0.792); Mfn1 (WT: 1 ± 0.32; APP/PS1: 0.56 ± 0.20; *p* = 0.321); Mfn2 (WT: 1 ± 0.35; APP/PS1: 0.89 ± 0.21; *p* = 0.815); OPA1 (WT: 1 ± 0.43; APP/PS1: 1.04 ± 0.32; *p* = 0.935); and ATP5A (WT: 1 ± 0.15; APP/PS1: 1.19 ± 0.30; *p* = 0.607; Supplementary Figures 1A, B). Consistent with the mPFC findings, we also found no significant difference in HIPP levels of Drp1 (WT: 1 ± 0.16; APP/PS1: 0.84 ± 0.17; *p* = 0.548); Mfn1 (WT: 1 ± 0.21; APP/PS1: 0.85 ± 0.05; *p* = 0.535); Mfn2 (WT: 1 ± 0.19; APP/PS1: 0.74 ± 0.11; *p* = 0.326); OPA1 (WT: 1 ± 0.14; APP/PS1: 0.88 ± 0.29; *p* = 0.741); and ATP5A (WT: 1 ± 0.19; APP/PS1: 0.90 ± 0.29; *p* = 0.801; Supplementary Figures 1C, D). Our data suggest that expression of mitochondrial fission/fusion proteins and ATP synthase is normal in the mPFC and HIPP of APP/PS1 mice at 1∼2 months of age.

**FIGURE 1:**
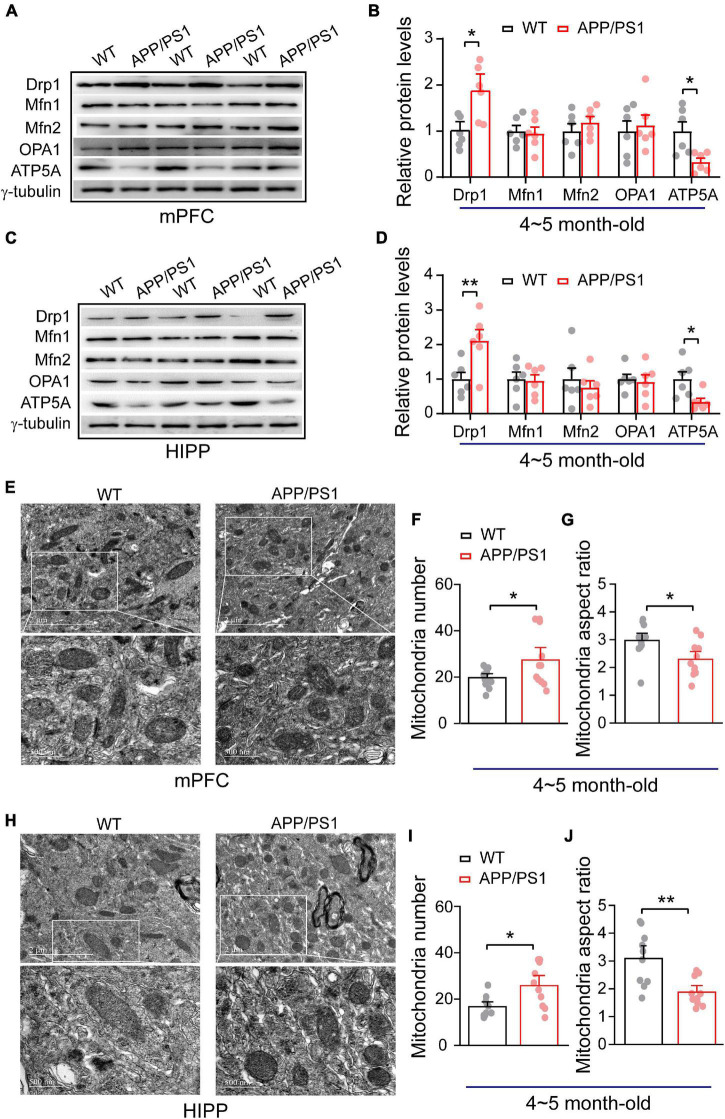
Alteration in mitochondrial fission, ATP synthase protein expression and mitochondrial morphology in the mPFC and HIPP of 4∼5-month-old APP/PS1 mice. **(A)** Representative immunoblotting images of mPFC extracts and **(B)** quantification of blots indicate significantly increased levels of Drp1 and decreased levels of ATP5A, whereas fusion proteins Mfn1, Mfn2, and OPA1 remain unchanged in APP/PS1 mice compared with age-matched WT mice (*n* = 6 mice per genotype; two repeats: two-sample *t*-test). **(C)** Representative immunoblotting images of hippocampus extracts and **(D)** quantification of blots indicate significantly increased levels of Drp1 and decreased levels of ATP5A, whereas fusion proteins Mfn1, Mfn2, and OPA1 remain unchanged in APP/PS1 mice compared with age-matched WT mice (*n* = 6 mice per genotype; two repeats; two-sample *t*-test). **(E)** Representative TEM images of mitochondrial morphology from mPFC of WT and APP/PS1 mice. **(F)** Increased mitochondrial number but **(G)** reduced mitochondrial aspect ratio in the mPFC of APP/PS1 mice than WT mice (*n* = 12 images for WT, 11 images for APP/PS1 mice; 3 mice per genotype: two-sample *t*-test). **(H)** Representative TEM images of mitochondrial morphology from HIPP of WT and APP/PS1 mice. **(I)** Increased mitochondrial number but **(J)** reduced mitochondrial aspect ratio in the HIPP of APP/PS1 mice than WT mice (*n* = 10 images for WT, 10 images for APP/PS1 mice; 3 mice per genotype: two-sample *t*-test). Each value represents the mean ± SEM, *p < 0.05, **p < 0.01.

Importantly, a balance of fission and fusion is crucial not only for mitochondrial morphology, but also for cell viability, synaptic function and neuronal morphology. We found identical fission/fusion and ATP synthase levels at 1∼2 months of age in APP/PS1 mice, so we asked whether alterations in these mitochondrial proteins occur later than this age. Considering that APP/PS1 mice exhibit a trend of increased soluble Aβ in cortex and HIPP at 4∼5 months-old (Jankowsky et al., 2004; Garcia-Alloza et al., 2006), we examined whether 4∼5 month-old APP/PS1 mice exhibit any alteration in these mitochondrial fission/fusion proteins. Western blotting was conducted in both mPFC and HIPP. In mPFC, we found significantly increased levels of fission protein Drp1 (WT: 1 ± 0.13; APP/PS1: 1.84 ± 0.24; *p* = 0.012), leaving levels of fusion proteins unchanged: Mfn1 (WT: 1 ± 0.12; APP/PS1: 0.94 ± 0.14; *p* = 0.782); Mfn2 (WT: 1 ± 0.16; APP/PS1: 1.18 ± 0.12; *p* = 0.384); OPA1 (WT: 1 ± 0.22; APP/PS1: 1.12 ± 0.22; *p* = 0.699). Notably, ATP5A levels decreased in APP/PS1 mice (WT: 1 ± 0.20; APP/PS1: 0.32 ± 0.08; *p* = 0.012; Figures 1A,B). The findings for HIPP reflected those for mPFC, revealing significantly increased levels of fission protein Drp1 (WT: 1 ± 0.19; APP/PS1: 2.10 ± 0.31; *p* = 0.013) and decreased ATP5A levels (WT: 1 ± 0.20; APP/PS1: 0.34 ± 0.10; *p* = 0.017) in APP/PS1 mice, while fusion protein levels were identical: Mfn1 (WT: 1 ± 0.20; APP/PS1: 0.94 ± 0.17; *p* = 0.852); Mfn2 (WT: 1 ± 0.32; APP/PS1: 0.75 ± 0.19; *p* = 0.525); OPA1 (WT: 1 ± 0.13; APP/PS1: 0.92 ± 0.20; *p* = 0.753; Figures 1C,D).

Given that mitochondrial morphology is regulated by fission and fusion (Franco et al., 2016; Lee et al., 2016). We found increased mitochondrial fission (Drp1) in APP/PS1 mice; therefore, we asked if this could result in abnormal mitochondrial morphology. Using TEM, we have examined mitochondrial morphology in mPFC and HIPP of 4∼5 month-old APP/PS1 mice. Our data analysis of mPFC indicates an increased number of mitochondria (WT: 19.75 ± 1.14; APP/PS1: 27.36 ± 3.58; *p* = 0.047) and decreased mitochondria aspect ratio (WT: 2.96 ± 0.17; APP/PS1: 2.29 ± 0.18; *p* = 0.014; Figures 1F,G) in APP/PS1 mice. Similarly, HIPP also exhibits abnormal mitochondrial morphology in APP/PS1 mice, such as an increased number of mitochondria (WT: 16.7 ± 1.39; APP/PS1: 25.8 ± 2.93; *p* = 0.011); and decreased mitochondrial aspect ratio (WT: 3.08 ± 0.30; APP/PS1: 1.87 ± 0.16; *p* = 0.002; Figures 1I,J).

### More Severe Alteration of Mitochondrial and Adenosine Triphosphate Synthase Protein Expression in Older Amyloid Precursor Protein/Presenilin 1 Mice

Aging is the primary risk factor for AD and is also associated with mitochondrial dysfunction, including impaired mitochondrial fission/fusion, biogenesis, and ATP production (Swerdlow et al., 2017; Hou et al., 2019). We next evaluated whether alterations in mitochondrial dynamics persist in older, i.e., 9∼10 month-old, APP/PS1 mice. Western blotting of mPFC extracts revealed an increased level of fission protein Drp1 (WT: 1 ± 0.06; APP/PS1: 2.16 ± 0.14; *p* < 0.001), but decreased levels of fusion proteins Mfn1 (WT: 1 ± 0.21; APP/PS1: 0.29 ± 0.11; *p* = 0.016) and OPA1 (WT: 1 ± 0.15; APP/PS1: 0.43 ± 0.06; *p* = 0.006), although no difference was found for Mfn2 (WT: 1 ± 0.06; APP/PS1: 0.83 ± 0.15; *p* = 0.361). Moreover, levels of ATP synthase protein ATP5A were decreased in mPFC (WT: 1 ± 0.11; APP/PS1: 0.40 ± 0.12; *p* = 0.005; Supplementary Figures 2A, B). A similar pattern was observed in HIPP, i.e., significantly increased levels of fission protein Drp1 (WT: 1 ± 0.20; APP/PS1: 3.51 ± 0.59; *p* = 0.002), but decreased levels of fusion proteins Mfn1 (WT: 1 ± 0.30; APP/PS1: 0.26 ± 0.06; *p* = 0.041), Mfn2 (WT: 1 ± 0.15; APP/PS1: 0.40 ± 0.11; *p* = 0.011) and OPA1 (WT: 1 ± 0.10; APP/PS1: 0.48 ± 0.04; *p* = 0.001), as well as decreased ATP5A levels (WT: 1 ± 0.13; APP/PS1: 0.49 ± 0.04; *p* = 0.004; Supplementary Figures 2C, D).

Given that aged APP/PS1 mice exhibit progressive development of Aβ plaques, which begins at 8 months of age (Garcia-Alloza et al., 2006), together with altered synaptic transmission and impaired memory (Mcclean and Holscher, 2014), we next asked whether aging worsened expression of mitochondrial proteins in AD model mice. Western blotting of proteins extracted from 12∼18 month-old APP/PS1 mPFC exhibited increased levels of fission protein Drp1 (WT: 1 ± 0.07; APP/PS1: 1.41 ± 0.04; *p* = 0.003), but decreased levels of fusion proteins Mfn1 (WT: 1 ± 0.12; APP/PS1: 0.38 ± 0.01; *p* = 0.002), Mfn2 (WT: 1 ± 0.05; APP/PS1: 0.66 ± 0.05; *p* < 0.001) and OPA1 (WT: 1 ± 0.08; APP/PS1: 0.57 ± 0.11; *p* = 0.010). Moreover, levels of ATP synthase subunit, ATP5A, also decreased (WT: 1 ± 0.04; APP/PS1: 0.67 ± 0.06; *p* = 0.002; Supplementary Figures 3A, B). HIPP, again, revealed significantly increased levels of fission protein Drp1 (WT: 1 ± 0.02; APP/PS1: 2.56. ± 0.14; *p* < 0.001), but decreased levels of fusion proteins Mfn1 (WT: 1 ± 0.03; APP/PS1: 0.66 ± 0.02; *p* < 0.001), Mfn2 (WT: 1 ± 0.02; APP/PS1: 0.69 ± 0.04; *p* < 0.001) and OPA1 (WT: 1 ± 0.01; APP/PS1: 0.61 ± 0.03; *p* < 0.001), as well as decreased levels of ATP synthase subunit, ATP5A (WT: 1 ± 0.06; APP/PS1: 0.65 ± 0.06; *p* = 0.002; Supplementary Figures 3C, D).

The above results suggest that 4∼5 months is the earliest age at which APP/PS1 mice exhibit alterations in proteins crucial for mitochondrial dynamics, abnormal mitochondrial morphology and energy metabolism, and that these effects persist in older mice (9∼10, 12∼18 month-old). We then focused on 4∼5 month-old APP/PS1 mice and age-matched WT controls in the following experiments unless otherwise indicated.

### Altered Dendritic Morphology in the Medial Prefrontal Cortex and Hippocampus

**FIGURE 2:**
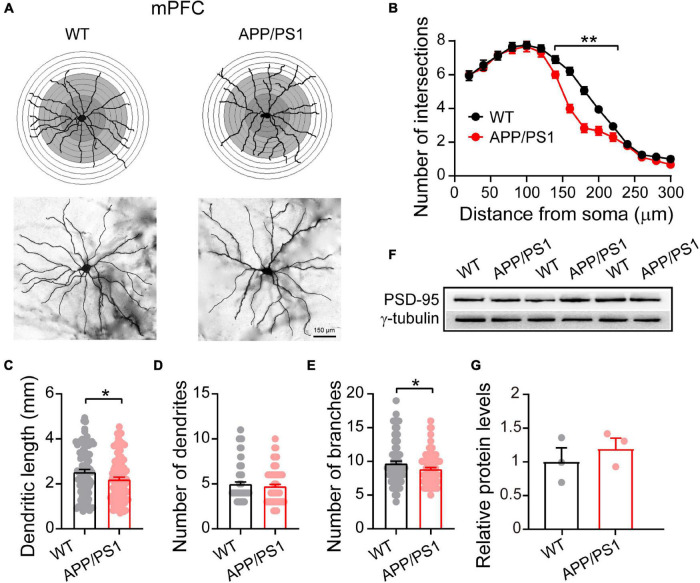
Altered dendritic morphology in the mPFC of 4∼5-month-old APP/PS1 mice. **(A)** Schematic photomicrographs of neurons with the allocation of dendrites between repeated 20 μm-spaced concentric rings. **(B)** APP/PS1 mice have fewer intersection points than age-matched WT mice (at a distance of 140–220 μm from the soma). **(C–E)** The dendrite length and number of branches of PFC neurons are significantly decreased in APP/PS1 mice compared with age-matched WT animals (*n* = 100 neurons from 4 animals per genotype; one-way ANOVA). **(F)** Representative immunoblotting images of PFC extracts and **(G)** quantification of blots indicate identical levels of PSD-95 in APP/PS1 mice compared with age-matched controls (*n* = 3 mice per genotype; two-sample *t*-test). Each value represents the mean ± SEM; *p < 0.05, **p < 0.01.

Dendritic morphology critically regulates the electrical properties of the neuron, with adult cortical neurons receiving around 15,000 synaptic inputs (Bianchi et al., 2013). To meet neuronal energy demands, normal mitochondrial fission and fusion are crucial as they enable mitochondrial transport within neurons from soma to dendrites and axons. Importantly, defects in mitochondrial function can lead to severe alteration in neuronal morphology, eventually resulting in the death of neurons (Sheng and Cai, 2012). The elevated Drp1 levels in 4∼5 month-old APP/PS1 mice, described above, make increased mitochondrial fragmentation likely, which in turn can alter neuronal morphology (Bertholet et al., 2016). We therefore examined the dendritic morphology of pyramidal neurons in the mPFC using Golgi-Cox analysis (Figure 2A) and found that 4∼5 month-old APP/PS1 mice had fewer intersections at a distance of 140–220 μm from the soma [*F*(_1,84_) = 485.113; *p* < 0.01; Figure 2B]. APP/PS1 mice also showed significantly reduced dendritic length [*F*(_1,199_) = 5.020; *p* = 0.026] and number of dendritic branches [*F*(_1,199_) = 4.620; *p* = 0.032], but no difference in the number of dendrites [*F*(_1,199_) = 0.743; *p* = 0.389] (Figures 2C–E).

Having uncovered neuronal morphological alterations, we next analyzed the expression of PSD-95 in the mPFC. PSD-95 is an important postsynaptic scaffolding protein that plays a crucial role in dendritic remodeling and synaptic development (Sweet et al., 2011). However, the results revealed unchanged levels of PSD-95 in the mPFC of 4∼5 month-old APP/PS1 mice (WT: 1 ± 0.18; APP/PS1: 1.19 ± 0.14; *p* = 0.463; Figures 2F,G), suggesting that the changes in neuronal morphology were not due to abnormalities in PSD-95 expression.

**FIGURE 3:**
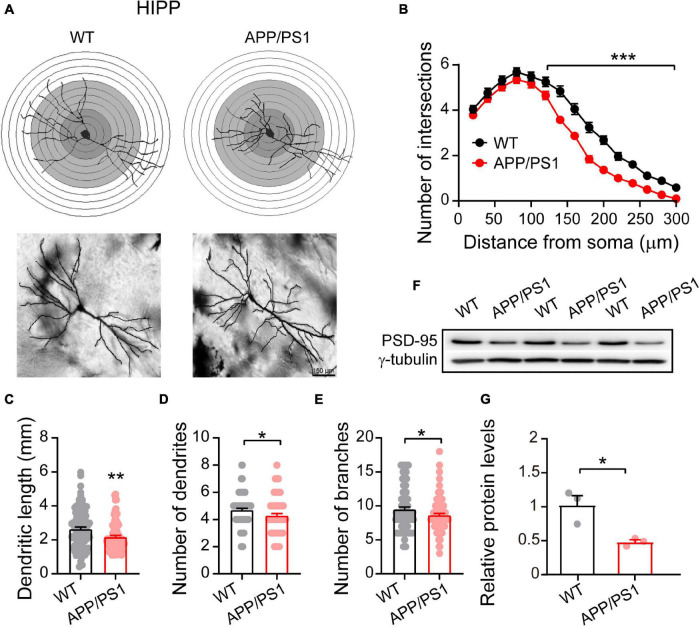
Altered dendritic morphology and decreased PSD-95 levels in the HIPP of 4∼5-month-old APP/PS1 mice. **(A)** Schematic photomicrographs of neurons with the allocation of dendrites between repeated 20 μm-spaced concentric rings. **(B)** APP/PS1 mice exhibit a reduced number of intersection points compared with age-matched WT (at a distance of 120–300 μm from the soma). **(C–E)** The dendrite length, number and branches of hippocampal neurons are significantly decreased in APP/PS1 mice compared with age-matched WT animals (*n* = 100 neurons from 4 animals per genotype; one-way ANOVA). **(F)** Representative immunoblotting images of HIPP extracts and **(G)** quantification of blots indicate significantly decreased levels of PSD-95 in APP/PS1 mice compared with age-matched WT mice (*n* = 3 mice per genotype; two-sample *t*-test). Each value represents the mean ± SEM; *p < 0.05, **p < 0.01, ***p < 0.001.

Research suggests that HIPP shrinkage may be an early sign of AD, because it occurs years before memory loss, and other symptoms, appear (Fang et al., 2019). Our Golgi-Cox analysis of HIPP pyramidal neurons showed that 4∼5 month-old APP/PS1 mice had fewer intersections between 120 and 300 μm from the soma [*F*_(1,98)_ = 439.813; *p* < 0.001; Figure 3B], and significantly reduced dendritic length [*F*_(1,199)_ = 10.239; *p* = 0.001], number of dendrites [*F*_(1,199)_ = 4.858; *p* = 0.028], and number of dendritic branches [*F*_(1,199)_ = 4.683; *p* = 0.031] (Figures 3C–E). Interestingly, in contrast to the situation in mPFC, we observed decreased levels of PSD-95 in HIPP of 4∼5 month-old APP/PS1 mice (WT: 1 ± 0.13; APP/PS1: 0.46 ± 0.03; *p* = 0.020; Figures 3F,G).

### Decreased Miniature Excitatory Postsynaptic Current Frequency in Pyramidal Neurons of Medial Prefrontal Cortex

**FIGURE 4:**
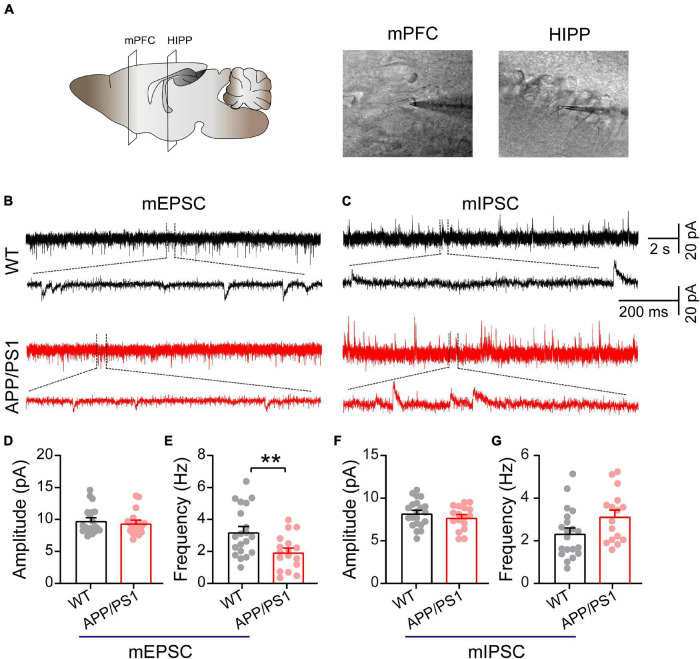
Decreased mEPSC frequency in pyramidal neurons of mPFC in 4∼5-month-old APP/PS1 mice. **(A)** Schematic of the mPFC and HIPP pyramidal neuron where whole-cell patch-clamp recording was performed. **(B)** Representative mEPSC and **(C)** mIPSC traces recorded in the mPFC. **(D,E)** Statistical analysis indicating significantly increased frequency but not amplitude of mEPSCs in APP/PS1 mice (frequency: *p* = 0.0072; amplitude: *p* = 0.5751; WT: *n* = 20 cells of 6 mice; APP/PS1: *n* = 17 cells of 6 mice). **(F,G)** Identical mIPSC frequency and amplitude in APP/PS1 mice (frequency: *p* = 0.0512; amplitude: *p* = 0.3179; WT: *n* = 21 cells of 6 mice; APP/PS1: *n* = 17 cells of 6 mice; two-sample *t*-test). Each value represents the mean ± SEM; **p < 0.01.

Mitochondria play an important role in presynaptic and postsynaptic neurotransmission through calcium buffering and a range of metabolic functions (Tang and Zucker, 1997; Devine and Kittler, 2018). Moreover, in neurons, fission uniquely facilitates the movement of mitochondria within axons and dendrites; disruptions of this movement due to alterations in the mitochondrial fission/fusion process specifically cause synaptic abnormalities and neuronal death (Shields et al., 2015; Devine and Kittler, 2018). Therefore, we determined whether mitochondrial dysfunction, as revealed by abnormal levels of mitochondrial proteins, might impair synaptic transmission in the mPFC and HIPP (Figure 4). Under the IR-DIC microscope, pyramidal neurons were identified by their typical triangular-shaped soma (Choy et al., 2018). The results showed unchanged mEPSC amplitude (WT: 9.80 ± 0.47 pA; APP/PS1: 9.43 ± 0.44 pA; *p* = 0.58; Figures 4B,D), but significantly decreased frequency (WT: 3.22 ± 0.43 Hz; APP/PS1: 1.95 ± 0.26 Hz; *p* = 0.0072; Figure 4E) in pyramidal neurons of mPFC in 4∼5-month-old APP/PS1 mice. In contrast, neither the amplitude nor the frequency of mIPSCs differed between the two groups (amplitude: WT: 8.22 ± 0.34 pA; APP/PS1: 7.73 ± 0.34 pA; *p* = 0.3179, frequency: WT: 2.35 ± 0.26 Hz; APP/PS1: 3.14 ± 0.3 Hz; *p* = 0.0512; Figures 4C,F,G). Moreover, mEPSC (*n* = 22 and 21 for WT and APP/PS1, respectively) and mIPSC (*n* = 23 and 22 for WT and APP/PS1, respectively) remained unchanged in the CA1 region of the HIPP in APP/PS1 compared to WT controls (data not shown).

### Abnormal Connectivity Between the Medial Prefrontal Cortex and Hippocampus

**FIGURE 5:**
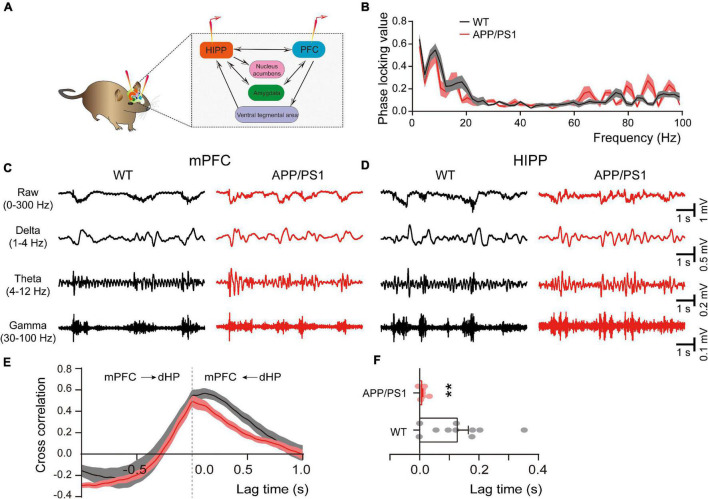
Altered connectivity between mPFC and HIPP of 4∼5 month-old APP/PS1 mice. **(A)** Schematic diagram showing *in vivo* dual-site LFP recording in the HIPP-mPFC. **(B)** The phase-locking curve between frequency ranges. **(C,D)** Representative traces of extracellular LFPs, as well as filtered delta, theta, and gamma oscillations, were recorded simultaneously in the mPFC and HIPP. **(E)** The LFP signals between mPFC and HIPP have approximately symmetrical cross-correlation values for positive (HIPP leading) and negative (mPFC leading) time lags in WT and APP/PS1, suggesting bidirectional communication between these two brain areas. **(F)** Quantification shows a significantly decreased cross-correlation value at positive time lag ranges between HIPP and mPFC in 4∼5 month-old APP/PS1 mice, indicating decreased synchronization between the two regions. WT: *n* = 10 mice; APP/PS1: *n* = 8 mice; two-sample *t*-test. Values represent the mean ± SEM, **p < 0.01.

The direct HIPP-PFC pathway originates from the CA1 region of the HIPP and subiculum, selectively projecting to the prelimbic mPFC and orbitomedial frontal cortex (Thierry et al., 2000; Preston and Eichenbaum, 2013), and is critically involved in working memory and social interaction behavior (Sun et al., 2020). We thus evaluated HIPP-PFC connectivity by dual-site extracellular recordings in the mPFC and HIPP (Figure 5A). Although similar phase locking was observed in both brain areas of WT and APP/PS1 mice (Figure 5B), cross-correlation analysis revealed a significantly decreased correlation between mPFC and HIPP (Figures 5E,F), indicating reduced bidirectional communication and synchronization between the two areas in APP/PS1 mice.

### Increased Gliosis Accompanied by Oxidative Stress

**FIGURE 6:**
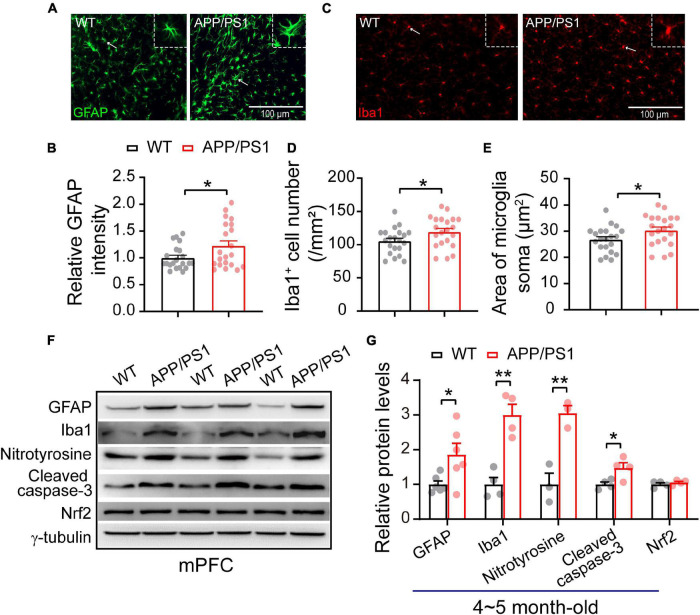
Increased neuroinflammation and oxidative stress in the mPFC of 4∼5 month-old APP/PS1 mice. **(A)** Immunofluorescent staining of GFAP in mPFC sections. **(B)** Higher levels of GFAP in the mPFC of APP/PS1 mice than in WT mice (7 slices/per mouse, 3 mice per genotype). **(C)** Immunofluorescent staining of Iba1 in mPFC sections. **(D,E)** Increased Iba1 + cell number and soma size, showing microglial activation, in the mPFC of APP/PS1 mice compared to WT mice (7 slices/per mouse, 3 mice per genotype). **(F,G)** Western blotting showing significantly increased levels of GFAP, Iba1, nitrotyrosine, and cleaved caspase-3 but unchanged Nrf2 in the mPFC of APP/PS1 mice compared to WT mice; *n* = 3–6 mice per genotype; two-sample *t*-test. Values represent the mean ± SEM, *p < 0.05, **p < 0.01.

**FIGURE 7:**
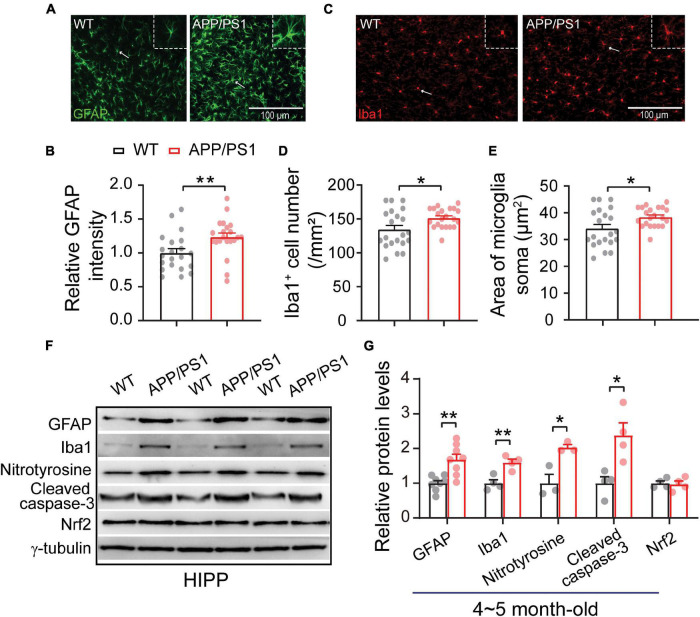
Increased neuroinflammation and oxidative stress in the HIPP of 4∼5 month-old APP/PS1 mice. **(A)** Immunofluorescent staining of GFAP in HIPP sections. **(B)** Increased levels of GFAP in the HIPP of APP/PS1 mice compared to WT mice (7 slices/per mouse, 3 mice per genotype). **(C)** Immunofluorescent staining of Iba1 in HIPP sections. **(D,E)** Increased Iba1 + cell number and soma size, showing microglial activation, in the HIPP of APP/PS1 mice compared to WT mice (7 slices/per mouse, 3 mice per genotype). **(F,G)** Western blotting showing significantly increased levels of GFAP, Iba1, nitrotyrosine, and cleaved caspase-3, but unchanged Nrf2 levels, in the HIPP of APP/PS1 mice compared to WT mice; *n* = 3–8 mice per genotype; two-sample *t*-test. Values represent the mean ± SEM, *p < 0.05, **p < 0.01.

The neuroinflammation ubiquitously observed in AD has emerged as a vital player in the progression of AD (Heneka et al., 2015; Da Mesquita et al., 2021; Leng and Edison, 2021). To examine whether astrogliosis occurs in 4∼5-month-old APP/PS1 mice, we conducted western blotting and immunofluorescent staining with anti-GFAP antibody, which is a standard marker of reactive astrocytes (Sofroniew, 2009). The staining and western blotting results revealed significantly enhanced levels of GFAP in both mPFC (WT: 1 ± 0.09; APP/PS1: 1.85 ± 0.32; *p* = 0.029; Figures 6A,G) and HIPP (WT: 1 ± 0.07; APP/PS1: 1.68 ± 0.15; *p* = 0.0013; Figures 7A,G) of APP/PS1 mice. Next, we asked if microglial activation, an indicator of neuroinflammation (Leng and Edison, 2021), occurs at this age in APP/PS1 mice. Utilizing anti-Iba1 antibody, widely used to detect active microglia under both normal and pathological conditions (Gheorghe et al., 2020), we observed a significantly increased number of Iba1-positive cells in mPFC (WT: 105.41 ± 4.22; APP/PS1: 119.29 ± 5.07; *p* = 0.042; Figure 6D) and HIPP (WT: 134.48 ± 6.05; APP/PS1: 151.36 ± 3.09; *p* = 0.017; Figure 7D) of APP/PS1 compared to WT mice. Moreover, significantly larger Iba1-labeled cell bodies occurred in the mPFC (WT: 26.76 ± 1.07; APP/PS1: 30.28 ± 1.28; *p* = 0.042; Figure 6E) and HIPP (WT: 34.14 ± 1.53; APP/PS1: 38.42 ± 0.78; *p* = 0.017; Figure 7E) of APP/PS1 mice. This microglial activation was further confirmed by western blotting (mPFC, WT: 1 ± 0.19; APP/PS1: 3.00 ± 0.30; *p* = 0.0014; Figures 6F,G) (HIPP, WT: 1 ± 0.09; APP/PS1: 1.59 ± 0.09; *p* = 0.005; Figures 7F,G), suggesting a remarkable enhancement of microglial activation, and likely subsequent neuroinflammation, at this age in AD mice.

Reciprocal interaction between neuroinflammation and dysfunctional mitochondria has been well documented (Van Horssen et al., 2019; Leng and Edison, 2021); these processes result in increased oxidative stress in the brain, leading to neuronal damage (Tonnies and Trushina, 2017; Misrani et al., 2021). Accordingly, we checked the expression of nitrotyrosine (a biomarker of oxidative stress) (Bandookwala and Sengupta, 2020) in the mPFC and HIPP of 4∼5-month-old APP/PS1 mice. Our results revealed a significantly increased level of nitrotyrosine in mPFC (WT: 1 ± 0.31; APP/PS1: 3.04 ± 0.21; *p* = 0.006) (Figures 6F,G) and in HIPP of APP/PS1 mice (WT: 1 ± 0.25; APP/PS1: 2.02 ± 0.08; *p* = 0.018) (Figures 7F,G). Cleaved caspase-3 induces synaptic dysfunction, neuronal loss and apoptosis in early AD (D’amelio et al., 2011). This caspase is responsible for the majority of proteolysis during apoptosis, and detection of cleaved caspase-3 is therefore considered a reliable marker for programmed cell death (Porter and Janicke, 1999; Lakhani et al., 2006). Neuroinflammation and oxidative stress can cause caspase-3 activation leading to apoptosis (Vince et al., 2018). We therefore tested levels of Cleaved caspase-3 and found these to be increased in mPFC (WT: 1 ± 0.06; APP/PS1: 1.47 ± 0.15; *p* = 0.029; Figures 6F,G) and in HIPP (WT: 1 ± 0.18; APP/PS1: 2.37 ± 0.36; *p* = 0.014; Figures 7F,G) of 4∼5-month-old APP/PS1 mice. However, levels of nuclear factor erythroid 2-related factor (Nrf2), which protects the brain from oxidative stress by upregulating the antioxidative defense pathway, inhibiting neuroinflammation, and maintaining protein homeostasis (Ahmed et al., 2017), remained unchanged in both mPFC (WT: 1 ± 0.04; APP/PS1: 1.05 ± 0.15; *p* = 0.36; Figures 6F,G) and HIPP (WT: 1 ± 0.06; APP/PS1: 0.96 ± 0.09; *p* = 0.079; Figures 7F,G).

### Impaired Social Interaction Memory

**FIGURE 8:**
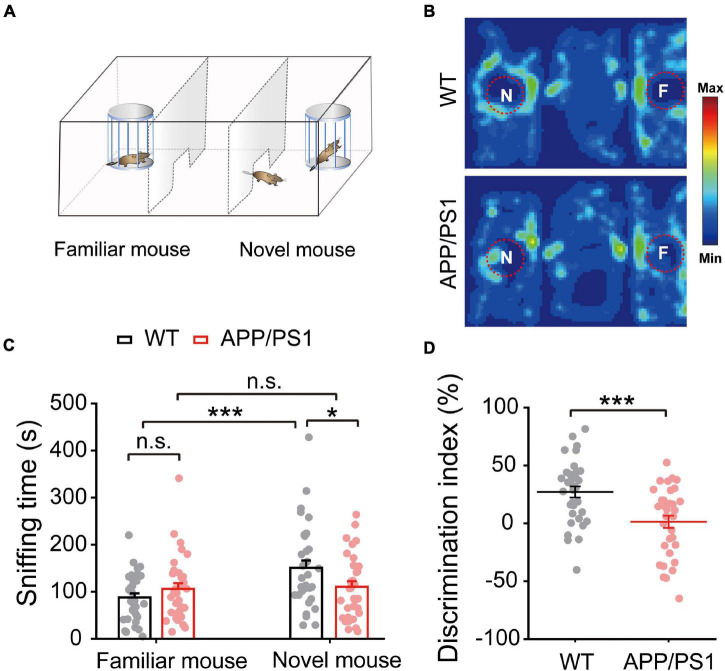
Impaired social interaction memory in 4∼5 month-old APP/PS1 mice. **(A)** Schematic diagram of the three-chamber social memory test setup, showing test mouse, familiar mouse chamber, and novel mouse chamber. **(B)** Representative heat map of a test mouse showing mouse activity during the test phase. **(C)** Time spent sniffing by test mouse in proximity to novel and familiar mice, showing that APP/PS1 mice spent less time than WT mice sniffing the novel mouse. **(D)** Decreased discrimination index of APP/PS1 mice compared to age-matched WT mice. Values represent mean ± SEM. WT: *n* = 33 mice; APP/PS1: *n* = 33 mice; one-way ANOVA, two-sample paired *t*-test. Values represent the mean ± SEM, *p < 0.05, ***p < 0.001.

Given that deficits in social communication, which requires normal HIPP and mPFC activity, occur in individuals with preclinical stage AD and mild cognitive impairment (MCI), we assessed the social interaction ability of 4∼5-month-old APP/PS1 and WT mice using a three-chamber social interaction test (Figure 8A). We found that APP/PS1 mice spent less time sniffing near the novel mouse chamber than WT mice (WT: 150.93 ± 15.44; APP/PS1: 110.49 ± 11.78; *p* = 0.041; Figures 8B,C). Moreover, data analysis of the discrimination index showed that APP/PS1 mice could not discriminate between familiar and novel mice as well as age-matched WT mice (WT: 27.16 ± 4.85; APP/PS1: 1.35 ± 5.26; *p* < 0.001; Figure 8D). Thus, although mice normally spend more time interacting with a novel mouse than a familiar one (Camats Perna and Engelmann, 2017), interaction time was decreased in APP/PS1 mice at the age tested.

## Discussion

**FIGURE 9:**
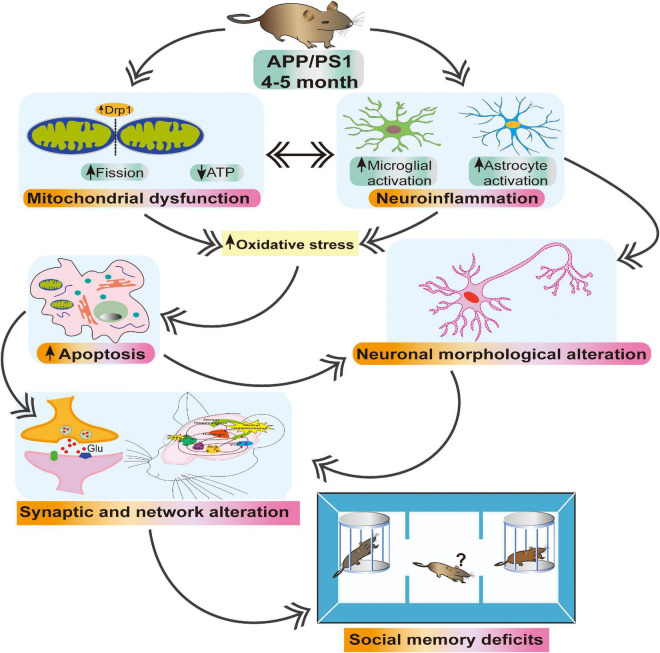
Schematic illustration depicting early alterations in APP/PS1 mice. Increased Drp1 and decreased ATP5A levels in 4∼5 month-old APP/PS1 (Figure 1) accompanied by astroglial activation (Figure 6), suggesting mitochondrial dysfunction and neuroinflammation likely contribute to oxidative stress, apoptosis (Figures 6, 7), and neuronal morphological alterations (Figures 2, 3). These alterations together lead to impaired synaptic activity (Figure 4) and network activity between mPFC and HIPP (Figure 5), which is associated with social interaction deficits (Figure 8).

It is becoming clear that the initial phase of AD begins years before the appearance of Aβ plaques and cognitive deficits (Frisoni et al., 2017; Styr and Slutsky, 2018). Recent research suggests that mitochondrial dysfunction (Hauptmann et al., 2009; Wang et al., 2020) and abnormal synaptic transmission (Selkoe, 2002; Styr and Slutsky, 2018) occur early at an early stage of disease progression, earlier than the emergence of histopathological or clinical abnormalities. Therefore, identifying the early mitochondrial and synaptic alterations at the prodromal phase of AD is of great importance for developing better diagnostic tools and more effective therapeutic interventions. Here, we show that, at 4∼5 months old (an age at which there are no obvious senile plaques) (Chen et al., 2021), APP/PS1 mice exhibit abnormal mitochondrial fission and morphology together with gliosis. These abnormalities are associated with neuroinflammation and oxidative stress (revealed by increased expression of nitrotyrosine), which may, in turn, cause activation of cleaved caspase-3 leading to apoptosis (Vince et al., 2018), as well as decreased levels of ATP5A (Terni et al., 2010). In combination, these alterations result in impairments of neuronal morphology, synaptic function and HIPP-mPFC network activity, which are associated with social interaction deficits (Figure 9). Together, the present study suggests that targeting mitochondrial dysfunction and neuroinflammation at an early stage of AD may slow down or prevent the pathogenesis of the disease.

Postmortem brains of AD patients exhibit defects in mitochondrial morphology and dynamics, and energy metabolism (Devi et al., 2006; Pickett et al., 2018). An imbalance between mitochondrial fission and fusion (increased fission and decreased fusion), as well as impaired ATP metabolism, occurs in AD patients and mouse models (Wang et al., 2009; Manczak et al., 2011, 2019; Kandimalla et al., 2018). Moreover, mitochondrial dysfunction, correlating with increased fragmentation, which is a common theme of neurodegeneration mainly in AD and is considered to be a fundamental early marker in disease progression (Wang et al., 2017, 2020; Oliver and Reddy, 2019). Our results reveal increased levels of mitochondrial fission protein, Drp1, and decreased levels of ATP synthase as early as 4∼5 months of age in APP/PS1 mice, suggesting that mitochondrial dysfunction is an early marker of AD pathogenesis (Figure 1). Inhibition of Drp1 ameliorates mitochondrial and synaptic dysfunction in 7 month-old APP/PS1 and 3 month-old CRND8 APP transgenic AD mice (Baek et al., 2017; Wang et al., 2017), as well as in other neurodegenerative diseases (Filichia et al., 2016). It is worth noting that the mitochondrial fission and fusion process regulate mitochondrial shape, mobility and transport. For example, abnormalities in mitochondrial fission and fusion and consequent changes in mitochondrial morphology influence mitochondrial mobility and distribution (Chen and Chan, 2009). Exogenous Aβ or overexpression of amyloid precursor protein (APP) cause profound fragmentation and impair mitochondrial transport in neuronal cultures (Wang et al., 2009; Manczak et al., 2011) and Drosophila model (Iijima-Ando et al., 2009). Mitochondrial membrane ATP synthase (F_1_F_0_ ATP synthase or Complex V) produces ATP from ADP in the presence of a proton gradient across the membrane which is generated by electron transport complexes of the oxidative phosphorylation (OXPHOS). The dysfunction of ATP synthase leads to disrupted OXPHOS and progressive ATP depletion. Compromised mitochondrial OXPHOS constitutes a characteristic mitochondrial deficit in AD brains, resulting in lowered ATP production, increased oxidative stress, and eventually cell death (Terni et al., 2010; Du et al., 2012). Given that reduced ATP synthase is repeatedly reported in the AD brain, our results also represent an early alteration in the energy-producing enzyme in APP/PS1 mice. Moreover, we also found abnormal mitochondrial morphology (increased fragmentation, increased number of mitochondria and reduced mitochondrial aspect ratio) in 4∼5 months-old APP/PS1 mice (Figure 1). Mitochondrial morphology depends on the balance between mitochondrial fission and fusion, and increased Drp1-induced mitochondrial fission could result in increased fragmentation and abnormal mitochondrial morphology (Detmer and Chan, 2007). Our results are in line with the increased number of mitochondria with Ca^2+^ overload in the brain of APP/PS1 (Xu et al., 2018; Calvo-Rodriguez et al., 2020). Given that a balance between mitochondrial fission and fusion is necessary for optimal energy metabolism, our findings suggest an age-related increase in mitochondrial fission and abnormal energy metabolism in the brain of APP/PS1 mice. These effects persist in older mice: we also found increased mitochondrial fission and decreased fusion and ATP synthase subunit levels in the mPFC and HIPP of 9∼10 month-old (Supplementary Figure 2) and 12∼18 month-old APP/PS1 mice (Supplementary Figure 3).

Reduced dendritic branching and length are common in the HIPP and cortical pyramidal neurons of AD patients and animal models (Tsai et al., 2004; Grutzendler et al., 2007). The morphology of dendritic branches is highly variable, and dynamic structures are continuously formed and eliminated throughout life (Grutzendler et al., 2002), a process that depends on the balance between mitochondrial dynamics and oxidative stress. We show that 4∼5 month-old APP/PS1 mice exhibit abnormal dendritic complexity and relative shortening of dendrite length and branching in both mPFC and HIPP (Figures 2, 3). These alterations in dendritic morphology may be due to increased oxidative stress and increased fission, which is consistent with the notion that mitochondrial dysfunction increases oxidative stress production in the brain, leading to neuronal damage (Li et al., 2004). PSD-95 is crucial for the maintenance of neuronal morphology (Sweet et al., 2011), and our western blotting results showed significantly reduced levels of PSD-95 in the HIPP of 4∼5 month-old APP/PS1 mice, which may be attributed, at least in part, to increased oxidative stress (Ansari et al., 2008). However, our results show no change in PSD-95 expression in the mPFC of 4∼5 month-old APP/PS1 mice. Consistent with the notion that AD patients and animal models exhibit region-specific alterations. Similarly, AD mice exhibit region-specific and age-related alterations in metabolic signaling, synaptic marker protein synaptophysin (presynaptic) and synaptic loss (Rutten et al., 2005; Gonzalez-Dominguez et al., 2014).

Besides energy production, mitochondria play an essential role in buffering intracellular Ca^2+^, and consequently these organelles are involved in maintaining and regulating synaptic transmission (Devine and Kittler, 2018). We found decreased mEPSC frequency in mPFC of 4∼5 month-old APP/PS1 mice (Figure 4), suggesting that a loss of mitochondria from these regions may impair synaptic transmission due to altered energy metabolism and regulation of intracellular Ca^2+^. Thus, a decline in mitochondrial function can occur decades before a clinical diagnosis of AD and may serve as a biomarker of AD risk, as well as a therapeutic target for the preservation of synaptic function (Caldwell et al., 2015). It is worth noting that, although both mPFC and HIPP showed abnormal expression of mitochondrial proteins and dendritic morphology, mEPSC frequency was reduced only in the mPFC and not in the HIPP. This decrease in mEPSC frequency suggests an early presynaptic alteration in the mPFC pyramidal neurons of 4∼5 month-old APP/PS1 mice. It has been shown that 4∼5 month-old APP/PS1 mice do not exhibit any HIPP-dependent memory loss or electrophysiological alteration at this age (Tabassum et al., 2019; Chen et al., 2021), and, indeed, hippocampal abnormalities do not appear until after 6 months of age in these AD-model mice (Viana Da Silva et al., 2016). We reason that AD pathogenesis may cause age-dependent and region-specific alterations in neuronal activities (Gengler et al., 2010; Oyelami et al., 2016; Chen et al., 2021), and that synaptic transmission in the mPFC is more vulnerable to neurodegeneration. Because mitochondrial morphology and function are crucial for synaptic transmission and function (Tang and Zucker, 1997; Devine and Kittler, 2018), we next investigated potential mitochondria-dependent mechanisms of synaptic transmission in pyramidal neurons of 4∼5-month-old APP/PS1 mice. Although 4∼5-month-old APP/PS1 mice exhibit increased Drp1 and decreased frequency of mEPSC in mPFC, incubation of the brain slices with Drp1 inhibitor, Mdivi-1, did not reverse the decline of mEPSC frequency (Supplementary Figure 4). Worth noting that inhibition of Drp1-induced mitochondrial fission protected synaptic damage in AD mice (Huang et al., 2015; Baek et al., 2017) and cell lines (Reddy et al., 2017); however, chronic administration is recommended. It is likely that mitochondria are located inside an intracellular compartment, and the drug molecule requires exact dosage and physicochemical properties to achieve therapeutic effects.

Functional connectivity between distant brain structures is fundamental in coordinating neuronal communication during sensory processing. Altered brain connectivity between brain regions occurs in patients with MCI and AD (Chan et al., 2013; Zheng et al., 2019). In the present study, we demonstrate significantly reduced coupling between mPFC and HIPP in 4∼5 month-old APP/PS1 mice (Figure 5), indicating an early HIPP-mPFC network alteration in AD. Our results are in agreement with recent opinion that early network dysfunction (altered neuronal activity and synchrony) contributes to neurodegeneration and AD pathogenesis (Frere and Slutsky, 2018; Mondragon-Rodriguez et al., 2019). Although the exact causes and mechanisms of network alterations have not been defined, our results suggest that mitochondrial dysfunction, neuroinflammation and oxidative stress may underlie these neuronal activity deficits.

Emerging evidence suggests that neuroinflammation has a causal role in the progression and pathogenesis of AD. In the present study, we show an increase in reactive astrocytes and microglial activation in the mPFC and HIPP of 4∼5 month-old APP/PS1 mice (Figures 6, 7). Astrocytic and microglial activation have been observed at the pre-plaque stage in 3∼6 month-old animal models of AD (Furman et al., 2012; Hanzel et al., 2014; Leng and Edison, 2021), and a human neuroimaging study reported increased microglial activation in individuals with MCI (Okello et al., 2009). We thus speculate that gliosis associated with neuroinflammation at an early age inappropriately engulfs synapses, resulting in early synaptic and network abnormalities.

We demonstrated an early impairment of non-cognitive AD-like symptoms, i.e., deficits in social interest, interaction, and communication in 4∼5 month-old APP/PS1 mice (Figure 8), similar to previous findings of deficits in social memory in 3∼6 month-old APP/PS1 mice (Filali et al., 2011; Pietropaolo et al., 2012). Given the critical role of HIPP and mPFC in mediating social behavior (Phillips et al., 2019), the present study suggests that mitochondrial dysfunction, together with increased neuroinflammation, and abnormal synaptic and network activity in the mPFC and HIPP, may underlie the social deficits observed in 4∼5 month-old APP/PS1 mice.

In conclusion, the most provocative finding of the present study is that age-related alterations in mitochondrial dynamics, which could be attributed, at least in part, to a number of pathways damaged by soluble Aβ, such as impairment of oxidative phosphorylation, elevation of ROS production, and interaction of Aβ with mitochondrial proteins (Pagani and Eckert, 2011; Mossmann et al., 2014; Pinho et al., 2014), excessive gliosis, and increased oxidative stress are early indicators of AD pathogenesis, contributing to impaired HIPP-mPFC activity and social interaction deficits in 4∼5 month-old APP/PS1 mice. Given that pre-amyloid deposition in humans begins at least two decades before the signs and symptoms of AD appear (Villemagne et al., 2018), and that targeting the early pre-amyloid phase may be an effective paradigm for the prevention of AD (Uhlmann et al., 2020), therapeutic interventions that improve mitochondrial function and reduce neuroinflammation represent promising strategies for slowing the progression or delaying the onset of this incurable disease.

## Data Availability Statement

The original contributions presented in the study are included in the article/Supplementary Material, further inquiries can be directed to the corresponding authors.

## Ethics Statement

The animal study was reviewed and approved by the Guangzhou University and South China Normal University Institutional Review Boards.

## Author Contributions

AM: project initiation, experimental design, western blotting, TEM statistical analysis, and manuscript writing. SiT: western blotting, TEM analysis, statistical analysis, figure generation, and manuscript writing. QH: LFP recording data analysis and figure generation. SuT: Golgi cox staining and data analysis. JJ: behavior and analysis and TEM. AA: immunostaining and analysis. XC and JWZ: whole-cell recording. JJZ: western blotting. SL and XF: behavior and analysis. CL: supervision. LY: guiding the experiments, funding acquisition, and critical revision.

## Conflict of Interest

The authors declare that the research was conducted in the absence of any commercial or financial relationships that could be construed as a potential conflict of interest.

## Publisher’s Note

All claims expressed in this article are solely those of the authors and do not necessarily represent those of their affiliated organizations, or those of the publisher, the editors and the reviewers. Any product that may be evaluated in this article, or claim that may be made by its manufacturer, is not guaranteed or endorsed by the publisher.
