# Supplementary material for: Mitochondrial Deficits With Neural and Social Damage in Early-Stage Alzheimer’s Disease Model Mice
Source: Front Aging Neurosci. 2021 Dec 10;13:748388. doi: 10.3389/fnagi.2021.748388 (PMC8704997; doi:10.3389/fnagi.2021.748388)
Supplement: Supplementary file 1 [file Data_Sheet_1.docx]

Supplementary Material

**
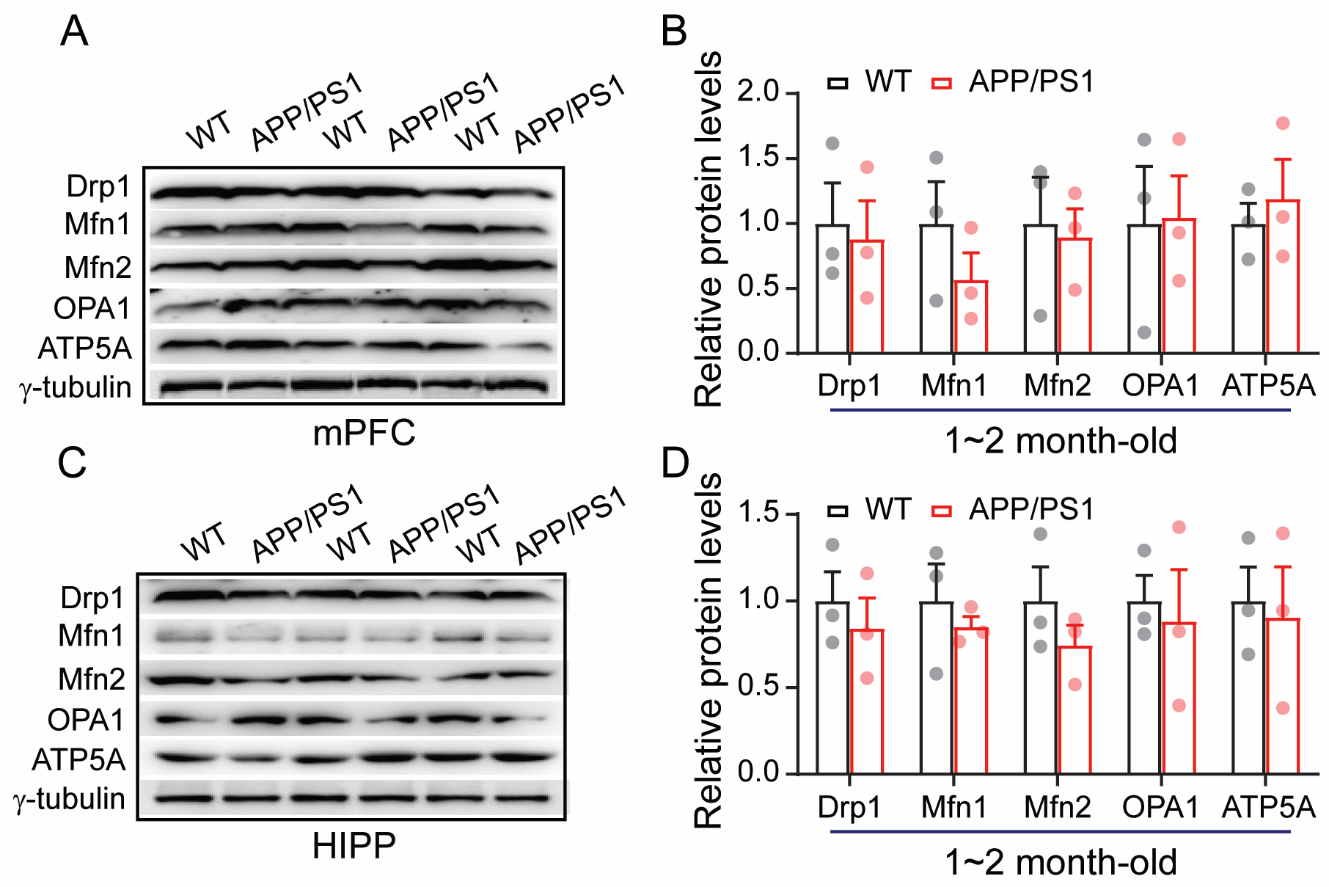
**

**Figure S1. Unchanged fission/fusion and ATP synthase protein expression in the mPFC and HIPP of 1~2-month-old APP/PS1 mice. (A)** Representative immunoblotting images of mPFC extracts and **(B)** quantification of blots indicate comparable expression levels of fission protein Drp1, fusion proteins Mfn1, Mfn2 and OPA1, and ATP5A in 1~2-month-old APP/PS1 mice compared with age-matched WT mice (n = 3 per genotype; two-sample *t*-test). **(C)** Representative immunoblotting images of HIPP extracts and **(D)** quantification of blots indicate comparable expression levels of fission protein Drp1, fusion proteins Mfn1, Mfn2 and OPA1, and ATP5A in 1~2-month-old APP/PS1 mice compared with age-matched WT mice (n = 3 mice per genotype; two-sample *t*-test).

**
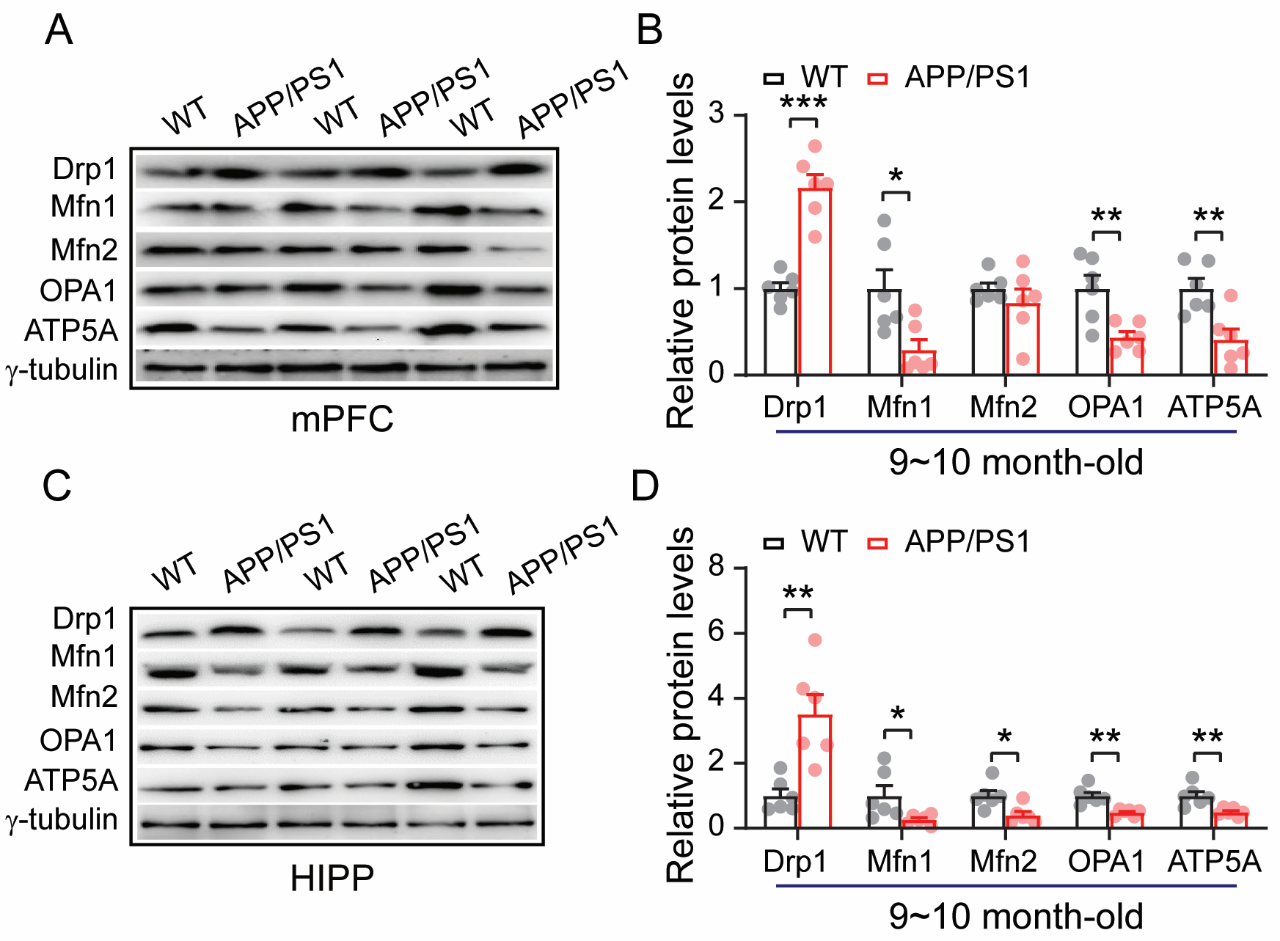
**

**Figure S2. Impaired fission/fusion and ATP synthase protein expression in the mPFC and HIPP of 9~10-month-old APP/PS1 mice. (A)** Representative immunoblotting images of mPFC extracts and **(B)** quantification of blots indicate significantly increased levels of Drp1 and decreased levels of Mfn1, OPA1 and ATP5A in 9~10-month-old APP/PS1 mice compared with age-matched WT mice (n = 6 mice per genotype; two repeats; two-sample *t*-test). **(C)** Representative immunoblotting images of HIPP extracts and **(D)** quantification of blots indicate significantly increased levels of Drp1 and decreased levels of Mfn1, Mfn2, OPA1 and ATP5A in 9~10-month-old APP/PS1 mice compared with age-matched WT mice (n = 6 mice per genotype; two repeats; two-sample *t*-test). Values represent mean ± SEM. **p* < 0.05, ***p* < 0.01, ****p* < 0.001.


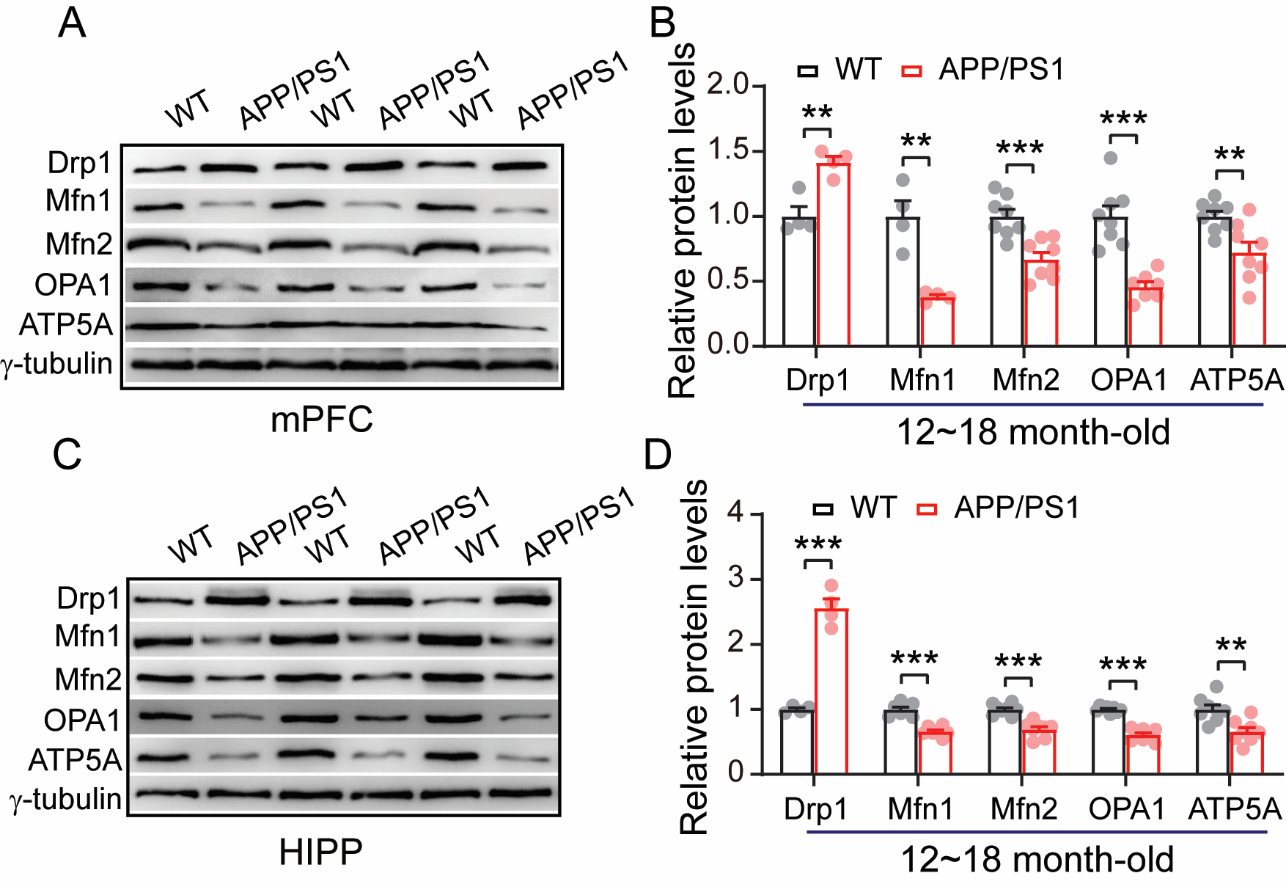


**Figure S3. Impaired fission/fusion and ATP synthase protein expression in the mPFC and HIPP of 12~18-month-old APP/PS1 mice. (A)** Representative immunoblotting images of mPFC extracts and **(B)** quantification of blots indicate significantly increased levels of Drp1 and decreased levels of Mfn1, Mfn2, OPA1, and ATP5A in 12~18-month-old APP/PS1 mice compared with age-matched WT mice (n = 4-8 mice per genotype; two repeats; two-sample *t*-test). **(C)** Representative immunoblotting images of HIPP extracts and **(D)** quantification of blots indicate significantly increased levels of Drp1 and decreased levels of Mfn1, Mfn2, OPA1, and ATP5A in 12~18-month-old APP/PS1 mice compared with age-matched WT mice (n = 4-8 mice per genotype; two repeats; two-sample *t*-test). Values represent mean ± SEM. ***p* < 0.01, ****p* < 0.001.


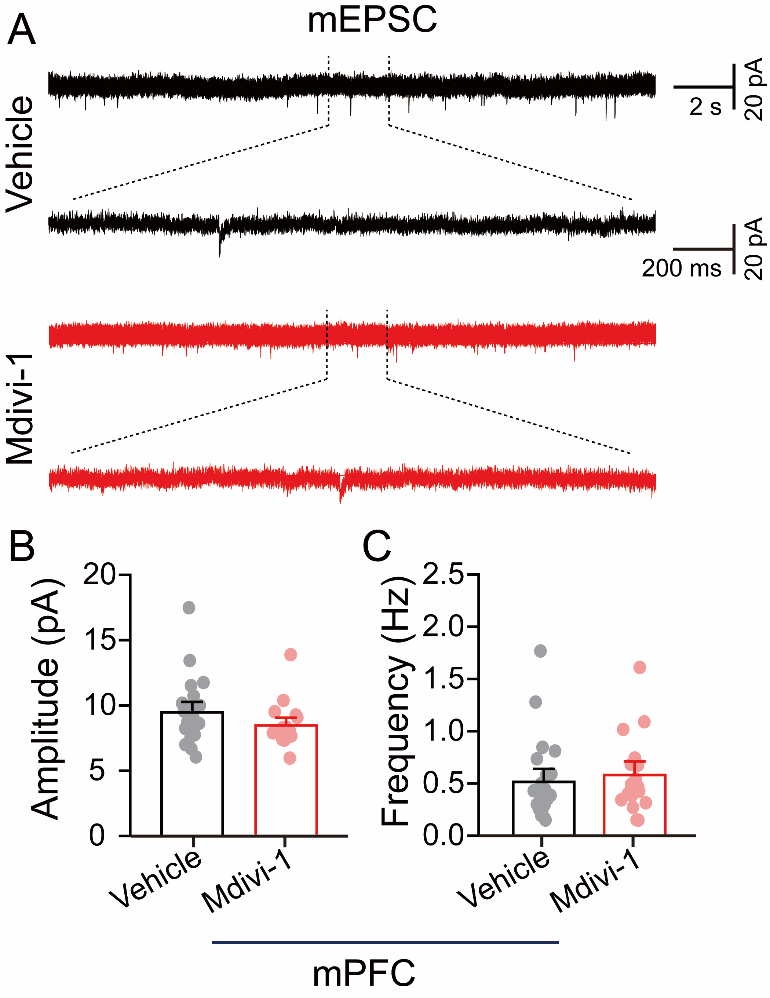


**Figure S4. Effects of mitochondrial Drp1 inhibitor Mdivi-1 on mEPSC of pyramidal neurons of mPFC in 4~5-month-old APP/PS1 mice. (A)** Representative mEPSC traces recorded in the mPFC. **(B-C)** Statistical analysis indicating no significant difference in frequency and amplitude of mEPSCs in vehicle or Mdivi-1 treated APP/PS1 mice (Vehicle: n = 23 cells of 3 mice; Mdivi-1: n = 18 cells of 3 mice). Values represents the mean ± SEM.
